# Upregulation of Beta4 subunit of BK_Ca_ channels in the anterior cingulate cortex contributes to mechanical allodynia associated anxiety-like behaviors

**DOI:** 10.1186/s13041-020-0555-z

**Published:** 2020-02-18

**Authors:** Huan Zhao, Qian Xue, Cong Li, Qingchuan Wang, Shichao Han, Yongsheng Zhou, Tao Yang, Yingli Xie, Hao Fu, Changbo Lu, Fancheng Meng, Ming Zhang, Yan Zhang, Xianglong Wu, Shengxi Wu, Min Zhuo, Hui Xu

**Affiliations:** 1 Department of Neurobiology and Collaborative Innovation Center for Brain Science, School of Basic Medicine, Fourth Military Medical University, Xi’an, 710032 China; 2 Center for Neuron and Disease, Frontier Institute of Science and Technology, Xi’an Jiaotong University, Xi’an, 710049 China; 3 Department of Phsyiology, University of Toronto, Toronto, Canada; 4 Department of Anesthesiology, Heze Municipal Hospital, Heze, 274031 Shandong China; 5 Shandong First Medcial University & Shandong Academy of Medical Sciences, Taian, 271000 Shandong China; 6 Key Laboratory for Space Bioscience and Biotechnology, School of Life Sciences, Northwestern Polytechnical University, Xi’an, 710072 China; 7 Department of Burns and Cutaneous Surgery, Xijing Hospital, Fourth Military Medical University, Xi’an, 710032 China

**Keywords:** Anterior cingulate cortex, Anxiety, Large-conductance Ca^2+^-activated potassium channel, Excitability, Synaptic transmission

## Abstract

The anterior cingulate cortex (ACC) serves as a critical hub for the anxiety and pain perception. The large-conductance Ca^2+^-activated potassium channels, or BK_Ca_ channels, are ubiquitously expressed throughout the central nervous system including the cingulate cortex. However, what changes of cortical BK_Ca_ channels undergo in the ACC remains unknown in pain-related anxiety. In the present study, a significant upregulation of synaptic and non-synaptic BK_Ca_ channel accessory β4 subunits in the ACC was accompanied with pain-associated anxiety-like behaviors in the chronic compression of multiple dorsal root ganglia (mCCD) of the rat. NS1619, an opener of BK_Ca_ channels, significantly rescued the alteration of fAHP and AP duration of ACC pyramidal neurons in mCCD rats. The mRNA expression of BK_Ca_ β4 subunits was extremely upregulated in the ACC after mCCD with the increased amount of both synaptic and non-synaptic BK_Ca_ β4 subunit protein. Meanwhile, NS1619 reversed the enhanced AMPA receptor-mediated spontaneous excitatory postsynaptic current (sEPSC) frequency and the attenuated PPR of ACC neurons in mCCD rats. Local activation of BK_Ca_ channels in the ACC reversed mechanical allodynia and anxiety-like behaviors. These results suggest that the upregulation of postsynaptic and presynaptic BK_Ca_ β4 subunit may contribute to neuronal hyperexcitability and the enhanced synaptic transmission in the ACC in neuropathic pain state, and then may result in anxiety-like behavior induced by neuropathic pain.

## Introduction

Recent advances in our understanding of altered central nervous system activity in pain-related anxiety patients have emerged from human brain imaging. Although many brain sites including the prefrontal and the insular cortices have been implicated in the regulated network of both affective pain and anxiety, the anterior cingulate cortex (ACC) serves as a critical hub for mood disorders, including anxiety and depression [1–4]. The stimulation of the ACC was sufficient to induce anxiety and depressive-like behaviors in naïve animals, while the ACC lesion prevented the pain anxiodepressive consequences of chronic pain without affecting the sensory mechanical allodynia [1]. Tons of evidence show that the ACC is involved in the pain-related negative affection [5]. However, the mechanisms underlying chronic pain related anxiety remain unclear in the ACC.

Clinical and experimental studies show that anxiety disorders and chronic pain often occur together. For example, anxious mood and other symptoms of anxiety were commonly seen in patients with chronic low back pain, cancer pain and diabetic neuropathy as well. Anxiety and depressive disorders are two common psychiatric disorders in patients with breast cancer [6]. Chronic pain could induce anxiogenic effects in complete Freund’s adjuvant (CFA) or neuropathic pain mice [7–9]. Greater pain intensity is correlated to more anxiety and depression in either spared nerve injury (SNI) mice or painful diabetic neuropathic patients [8, 10, 11]. A more recent study reported that cohabitation with a conspecific animal with chronic pain could potentially trigger anxiety in mice [12]. In turn, these negative emotional disorders aggravate pain perception. The patients with chronic back pain and pain-related anxiety are likely to have a tendency to overpredict pain events [13]. Previous studies also observed that anxiety might worsen pain perception [14–16]. The long-lasting anxiety exacerbated hypersensitive pain behaviors in the formalin rat model [17]. The interaction between chronic pain and anxiety is still not well understood.

BK_Ca_ channels are widely expressed throughout various tissues and cells in the central nervous system [18, 19], and play important roles in many physiological processes, including muscle contraction, circadian rhythm and hearing [20–22]. BK_Ca_ channels are tetramers characterized by a pore-forming α subunit containing seven transmembrane segments alone [23], or associated with accessory β subunits [20, 24]. Every β subunit has a specific tissue distribution and that it modifies channel kinetics as well as their pharmacological properties and the apparent Ca^2+^ sensitivity of the α subunit in different ways [23, 25–27]. Among the subunits of BK_Ca_ channels, α, β2, β4 types are predominantly expressed in central neurons [19]. The opening of BK_Ca_ channels allows rapid efflux of potassium ions, which effectively hyperpolarizes membrane potential, regulates membrane excitability [28, 29]. Recent work reported the downregulation and the decrease of BK_Ca_ channels in DRG and amygdala neurons in neuropathic pain and stress-induced anxiety [30–32], suggesting the possible functional involvement of BK_Ca_ channels in pain and anxiety. However, whether cortical BK_Ca_ channels undergo neuronal plastic changes in pain-related anxiety remains unclear. Our preliminary results showed a marked anxiety-like behavior in the newly established model of the chronic compression of multiple dorsal root ganglia (mCCD) [33]. Therefore, the aim of our present study was to investigate possible molecular mechanisms underlying pain-associated anxiety-like behaviors in the mCCD model at cortical level.

## Materials and methods

### Animals and mCCD model

All experiments were conducted on male Sprague-Dawley rats (weighing 150–200 g, 7–8 weeks old) purchased from the Laboratory Animal Center of Fourth Military Medical University (FMMU), Xi’an, Shaanxi Province, China. All animals were housed under a 12 h dark–12 h light cycle with food and water provided ad libitum (temperature 22–26 °C, air humidity 40–60%).The rats were housed in our labortary for at least 7 days before the initiation of the experiments. Rats were deeply anesthetized with an intraperitoneal (i.p.) injection of sodium pentobarbital (50 mg/kg body weight). All manipulations were done on the left side of the spinal column. Special care was paid to prevent infection and to minimize the influence of inflammation. The hair of the rats’ lower back was shaved and the skin was sterilized with 0.5% iodine tincture. Sterile surgical instruments were used. With the rats in a prone position, an incision was made along the midline of the back at the L2 and L6 spinal level. Following separation of the paraspinal muscles from the transverse process, the L3–5 intervertebral foramina were exposed. L-shaped rods made of hollow stainless steel (4 mm in length and 0.5–0.8 mm in diameter) were carefully inserted into the L3, L4 and L5 foramina to compress the DRGs. All behavioral testing occurred between 09.00 and 12.00 h on the designated day of experiment. All experimental protocols were approved by the Institutional Animal Care and Use Committee of FMMU and animals were maintained and cared for in line with the guidelines set forth by the International Association for the Study of Pain.

### Pain behavioral tests: mechanical allodynia

Paw withdrawal thresholds to mechanical stimulation were assessed as described [34] using Von-Frey filaments (Stoelting Corporation, USA). Animals were habituated to the behavioral test environment for 5–7 consecutive days before the testing. Mechanical allodynia was determined on ipsilateral and contralateral sides at postoperative 1, 3, 5, 7, 10 and 14 days. Animals were placed in plastic cages with a wire mesh floor. To test for the tactile threshold required to evoke withdrawal of the stimulated paw, von Frey filaments with different bending forces (2–15.0 g) were applied perpendicularly to the plantar part of the hindpaw in an ascending order. Each filament was applied 5 times to its minimum bending force, and a paw withdrawal threshold was defined as three positive responses. To avoid potential tissue damage, the cut-off threshold was assigned as 15.0 g-force.

### Rotator rod

Functional motor impairment was evaluated by the accelerating rotator rod test. A 5-min activity test was performed for each rat. Subjects received 5 consecutive training trials on the rotate rod, as previously described [35]. The rod accelerated from 4 rpm to 40 rpm over a 5-min period, and the latency (in seconds) for the animal to fall off the drum was recorded. The rotator rod is covered with fine grit sandpaper to provide a uniform surface and to reduce slipping [36]. The rotate rod was interfaced to a computer that collected the time each subject remained on the rod up to a maximum time of 360 s. After the 5 training trials were complete, averages of the last 3 trials were calculated and animals which failed to meet a criterion of 7 s were given additional training trials until their final 3 training trials averaged 7 s. The rats were trained on the rotarod for three consecutive days. The motor activity on the rotate rod was testing on postoperative day 7, and the mean latency for the animal to fall off the drum in the three test trials was recorded.

### Elevated plus maze

The elevated plus maze was a cross-shaped platform positioned 80 cm above the floor with two open arms (50.17 cm ×  10.8 cm) and two closed arms (50.17 cm ×  10.8 cm × 40.01 cm) on opposing sides of a central square platform (10.8 cm ×  10.8 cm). It is a well-established rodent test used to characterize anxiety-like behaviors. Rats were allowed to habituate to the testing room for 2 days before the test, and were pre-treated with gentle handling two times per day to eliminate their nervousness. For each test, individual animals were placed on the centre square, facing an open arm, and allowed to move freely for 5 min. Mice were videotaped using a camera fixed above the maze and analyzed with a video-tracking system. The number of entries and time spent on each arm were recorded. The elevated plus maze was cleaned by 70% ethanol after each rat to remove any possible olfactory cues.

### Open field Test

The open field apparatus recorded as a measurement for locomotor activities was a black square box (90 cm ×  90 cm × 60 cm). At the start of the test, each rat was placed at the centre of the box and allowed to explore the field freely for 15 min, during which its behavior was recorded by a video camera mounted above the maze. The field was divided into nine equal segments in the analyzed system. Anxiety-like behavior was determined by measuring the percentage of time spent in the center of the open field. The field was cleaned by 70% ethanol to remove any possible olfactory cues between each animal. An experimenter blind to treatment groups handled the animals and analyzed the video recordings.

### Quantitative (QT) real-time RT-PCR

mRNA expression of α and β (1–4) subunits of BK_Ca_ channels was evaluated by reverse transcription PCR. Total RNA was extracted from the ACC and the insular cortex and purified using RNAiso Plus and a Total RNA Isolation Kit (Takara, Otsu, Shiga, Japan).

The OD260/280 ratio of the RNA samples was measured, and the samples with a ratio of 1.8–2.0 were used for reverse transcription. In total, 0.5 mg RNA was reverse-transcribed to cDNA by using Prime Script™ RT Reagent Kit (Takara). The reactions were performed at 37 °C for 15 min and then 85 °C for 5 s. Quantitative RT-PCR analyses were performed in triplicate by using SYBR® Premix Ex Taq™ Kit (TaKaRa) and detected by using a Bio-Rad iQ5 Multicolor Real-Time PCR Detection System (Bio-Rad, Hercules, CA, USA). The data were normalised to β-actin, and the comparative cycle threshold (Ct) method (2-ΔΔCt) was used to calculate the relative quantity of target mRNAs.

Primers were designed by using the software Primer-BLAST developed at the National Center for Biotechnology Information (NCBI, Bethesda, MD, USA) and based on the software Primer 6. To check any possible genomic contamination of cDNA samples, each couple of primers was separated by at least one intron (minimum size of 1000 base pair) on the corresponding genomic DNA. The primer sequences were listed in Table 1.

**Table 1:** Sequence of primers used for RT-PCR

Gene	Accession no.	Sequence of; F, forward primer; R, reverse primer	Region	Product
*β-actin*	Rat	F:5′-ATGGTGGGTATGGGTCAGAAGG-3′	208–472	265 bp
	NM-031144	R: 5′-TGGCTGGGGTGTTGAAGGTC-3’		
*BK*_*Ca*_*αsubunit*	Rat	F: 5′- CAGCACTCCGCAGACACTA-3’	3774–3879	106 bp
	NM-031828.1	R:5′- ATCACCATAACAACCACCATCC-3’		
*BK*_*Ca*_*β1 subunit*	Rat	F:5′-CGCCATCACTTACTACATCCT − 3’	394–682	289 bp
	019273.1	R:5′-GACCTTCTTCACATCAACCAA-3’		
*BK*_*Ca*_*β2 subunit*	Rat	F: 5′-CGGACCTCTTCATCTTACAGAC-3’	22–210	189 bp
	176,861.1	R:5′-CAGCAGTGTGATTCCCAGTAG − 3’		
*BK*_*Ca*_*β3 subunit*	Rat	F:5′-CGCTCTGATTGTTGGTTTGGT − 3’	899–1059	161 bp
	001104560.2	R:5′-CTCTGTTCCTTCCCTGACTCTT-3’		
*BK*_*Ca*_*β4 subunit*	Rat	F:5′-CATCCTGTCGCTCTTCATCTTC-3’	102–335	234 bp
	023960.2	R: 5′ -TGGTGCTGGTCGCTGTGTA-3’		

*RT-PCR* real-time polymerase chain reaction, *BK*_*Ca*_ large-conductance, calcium-activated potassium

The following thermal cycle condition was employed to reverse-transcribe the mRNA: an initial denaturation at 95 °C for 30 s, followed by 40 cycles of denaturation at 95 °C for 5 s, and annealing at 60 °C for 30 s were performed.

### Western blot analysis

Western blot analysis was performed as described previously [32]. Tissue samples from the bilateral ACC were dissected from the brain slices under the anatomical microscope. 30 μg of total proteins were separated by electrophoresis on SDS-PAGE and then transferred to a polyvinylidene difluoride membrane (Invitrogen). The synaptosome was digested to yield an insoluble PSD-enriched (synaptic) membrane fraction and a soluble non-PSD enriched (peri/extrasynaptic and presynaptic) membrane fraction [37–39]. A clear separation of PSD and non-PSD membranes was collected as our previous study [39].

Membranes were incubated with primary antibody against BK_Ca_ α subunits (1:400, Alomone Labs, Jerusalem, Israel; product no., APC-021), BK_Ca_ β4 subunit (1:200, Alomone Labs, Jerusalem, Israel; product no., APC-061) or β-actin (1:1000, Sigma) as loading control overnight at 4 °C. Then the membranes were incubated with horseradish peroxidase-conjugated secondary antibodies (anti-rabbit IgG) for 1 h at 37 °C. The density of each protein band on the membrane was scanned using a FluroChem FC system (Alpha Innotech, San Jose, California, USA) and is presented as a densitometric ratio between the protein of interest and the loading control.

### Whole-cell patch-clamp recording

The animals were killed by being rendered unconscious by 4% isoflurane in air and then killed by cervical dislocation. Brain slices (300 μM) containing the ACC were cut at 4 °C with a vibratome in oxygenated artificial CSF containing (in mM): 124 NaCl, 2.5 KCl, 25 NaHCO_3_, 2 CaCl_2_, 2 MgSO_4_, 1 NaH_2_PO_4_, and 20 D-glucose, pH 7.4. For electrophysiology, brain slices were transferred to a submerged recovery chamber with oxygenated ACSF at room temperature. After 1 h of the recovery, slices were placed in a recording chamber on the stage of an Olympus microscope with infrared digital interference contrast optics for visualization of whole-cell patch-clamp recordings. The recording pipettes (3–5 MΩ) were filled with a solution containing (in mM) 145 K-gluconate, 5 NaCl, 1 MgCl_2_, 0.2 EGTA, 10 HEPES, 2 Mg-ATP, 0.1 Na3-GTP, and 10 phosphocreatine disodium (adjusted to pH 7.2 with KOH). For firing rate recording, Interneurons and pyramidal neurons were identified by their different firing patterns and morphology. The typical firing pattern of pyramidal neurons showed significant firing frequency adaptation, whereas interneurons showed fast-spiking APs followed by pronounced hyperpolarization, lower rheobase current and higher input resistance [40]. In the present study, layer II/III pyramidal neurons in the ACC were recorded. Access resistance 15–30 MΩ was considered acceptable. Data were discarded if access resistance changed > 15% during an experiment. Data were filtered at 1 kHz, and digitized at 10 kHz.

### Passive membrane properties

Off-line analysis was performed using Clampfit version 10.4 (Axon Instruments). Resting membrane potential (RMP) was the low-pass readout of the electrode amplifier and was not corrected for liquid junction potential (~ 12 mV) after terminating the recording. The membrane potential was measured immediately after establishing the whole cell configuration. Only neurons that had a resting membrane potential more negative than − 60 mV were further investigated.

### Active membrane properties and firing patterns

Action potentials (APs) were detected in response to suprathreshold current injections from a holding potential around − 60 mV. Depolarizing currents of − 100~480 pA (500ms duration) were delivered in increments of 20 pA until an AP was evoked. The rheobase was defined as the minimum current required to evoke an action potential. The AP voltage threshold (Vthreshold) was defined as the first point on the rising phase of the spike at which the change in voltage exceeded 50 mV/ms. The spike amplitude was quantified as the difference between the Vthreshold and the peak voltage. The duration of the AP was measured at the threshold voltage. The spike width was measured at 1/2 of the total spike amplitude (measured from the Vthreshold level). The time to the peak of fast component of the after hyperpolarization (fAHP) was estimated as the time from the peak of the action potential to the most negative voltage reached during the fAHP (defined as the peak of fAHP). The amplitude of fAHP was estimated as the difference between the Vthreshold and the peak of fAHP. The waveform characteristics of the action potentials recorded from neurons of sham and mCCD rats, i.e., rise time, rise slope, decay time and decay slope, were determined using Clampfit10.4 software (Axon Instruments). The properties of firing patterns and hyperpolarizing responses were analyzed from voltage response to injected current pulses.

In BK_Ca_ current experiments, TTX (1 μM), apamin (200 nM) and 4-AP (10 mM) were routinely added to extracellular solutions to block action potentials and minimize contamination by small-conductance, calcium-activated K^+^ (SK) currents [41] and voltage-dependent K^+^ (K_V_) currents, respectively [42, 43]. In voltage-clamp mode, macroscopic outward currents were elicited from a holding potential of − 60 mV, stepping to + 40 mV for 400 ms in 10 mV increments. BK_Ca_ currents were isolated by application of paxilline (10 μM), an antagonist of BK_Ca_ channels [44, 45], by subtracting currents in the presence of paxilline from the initial current.

### ACC cannulation and microinjection

Rats were anesthetized by intraperitoneal injections of sodium pentobarbital (40mg / kg). Rat heads were secured on a stereotaxic frame, and 24 gauge guide cannulae were implanted bilaterally into the ACC (0.5 mm anterior to bregma, ±0.4 mm lateral from the midline, 2.5 mm beneath the surface of the skull). The rats were given 1 week to recover after the cannula implantation. The 30 gauge injection cannula used was 0.1 mm lower than the guide cannula. For the microinjection, the animals were placed individually in an induction chamber, and anaesthesia was induced with 2.5% isoflurane (RWD, Shenzhen, China) in 100% oxygen with a delivery rate of 0.5 l/min until loss of righting reflex. Anaesthesia was then maintained with 1.5% isoflurane in 100% oxygen with a flow of 0.5 l/ min delivered by a face mask. The selective BK_Ca_ channel agonist NS1619 (10 μM, 0.5 μl) (Tocris) was infused into each side of the ACC at a rate of 0.5 μl/min; an equivalent volume of ACSF (0.5 μl) was used as a control using a syringe driven by an infusion pump (RWD, Shenzhen, China). After infusion, the cannula was left in place for an additional 2 min to allow the solution to diffuse away from the cannula tip. The injection sites were confirmed at the end of all experiments, and sites outside of the ACC region were excluded from the study. Elevated plus maze, open field tests and mechanical threshold were then reassessed 30 min after the microinjection of either ACSF or NS1619 in the ACC within 7–10  days after the compression of multiple DRG.

### Chemicals

(1,3-dihydro-1-[2-hydroxy-5-(trifluoromethyl)-phenyl]-5-(trifluoromethyl)-2H-benzimidazol-2-one), NS1619 (Tocris), a specific BK_Ca_ channel opener; Paxilline (alomone), a BK_Ca_ channel blocker; NS1619 and paxilline were both dissolved in dimethysulfoxide (DMSO), and then diluted in ACSF to the final concentration of 10 μM.

### Statistical analysis

Statistical analyses were performed by using SigmaStat 3.5 and GraphPad Prism 6 softwares. All data sets were tested for normality for t-test, and if normality test (Shapiro–Wilk) failed, Mann-Whitney Rank Sum test was used. Results are expressed as mean ± s.e.m. *p* < 0.05 is considered as a significant change. The two-way repeated-measure analysis of variance (ANOVA) was used to compare the differences between groups in the pain behavior testing and to analyze BK currents in sham and mCCD groups.

## Results

### Enhanced anxiety-like behaviors in mCCD rats

**Fig. 1:**
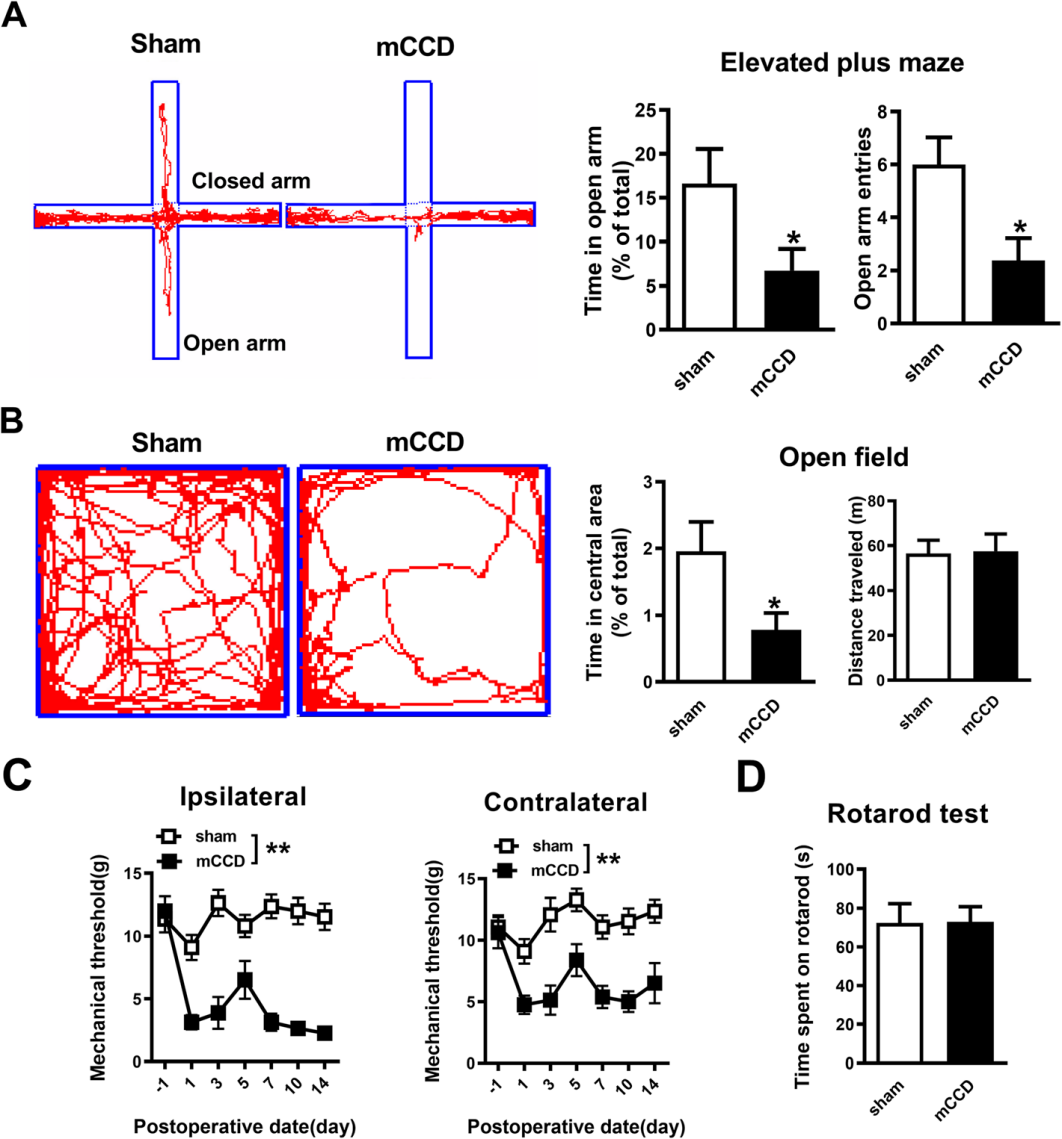
Enhanced anxiety in neuropathic pain rats following mCCD. **a,** Representative traces show the movement of sham and mCCD rats in elevated plus maze for 5 mins, mCCD rats (*n* = 13) spent less time in the open arms of elevated plus maze and showed a decrease in open arm entries compared with sham rats (*n* = 13). **b,** Representative traces show the movement of sham and mCCD rats in the open field test over a period of 15 min, mCCD rats (n = 13) spent significantly less time in the central zone in the open field test compared with sham rats (n = 13). Total distance travelled in the open field test was not changed compared with sham rats. **c**, The time course of ipsilateral and contralateral hind paw mechanical withdrawal thresholds after mCCD (sham group: *n* = 11, mCCD group: *n* = 8). The ipsilateral and contralateral hind paw withdrawal thresholds were significantly decreased below baseline on the 1st postoperative day and reached their lowest peak during the 7th to 14th postoperative day. **d,** Motor performances on the accelerating rotarod of mCCD rats (mCCD: *n* = 7, sham: n = 7). ^*^p < 0.05, ^**^p < 0.01 compared with that of sham group

To explore the pain-associated anxiety in mCCD rats, the rats were subjected to elevated plus maze (EPM) and open field tests on postoperative day 7–14. In the elevated plus maze, the increase of the time spent in the open arms is interpreted as a measure of decreased anxiety-like behaviors. The mCCD rat exhibited significantly lower entries and less time in the open arms than sham rats (Fig. 1a). The mCCD rat spent less time in the center of the open field (Fig. 1b), which is interpreted as anxiety-like behaviors [46]. Because the open field test can also be used as a measure of overall activity, we measured the total distance traveled in the open field. There was no difference in the total distance between mCCD and sham rats (Fig. 1b). The mechanical pain threshold was examined on days 1, 3, 5, 7, 10 and 14, postoperatively, in mCCD and sham rats. This increase in mechanical sensitivity was reflected in the large bilateral decrease in the hind paw withdrawal thresholds (defined as the minimum bending force required to elicit 50% response incidence) after mCCD. Peak of mechanical hypersensitivity occurred between 7 and 14 d after mCCD (Fig. 1c). However, neither the time spent on accelerating rotator rod nor the total distance travelled in the open field tests changed compared with those of sham group (Fig. 1d and b), showing that there was no difference in locomotor activity between mCCD and sham groups. These data demonstrate that neuropathic pain enhances anxiety-like behaviors in mCCD rats.

### Elevated excitability of ACC pyramidal neurons in mCCD rats (Table 2)

**Fig. 2:**
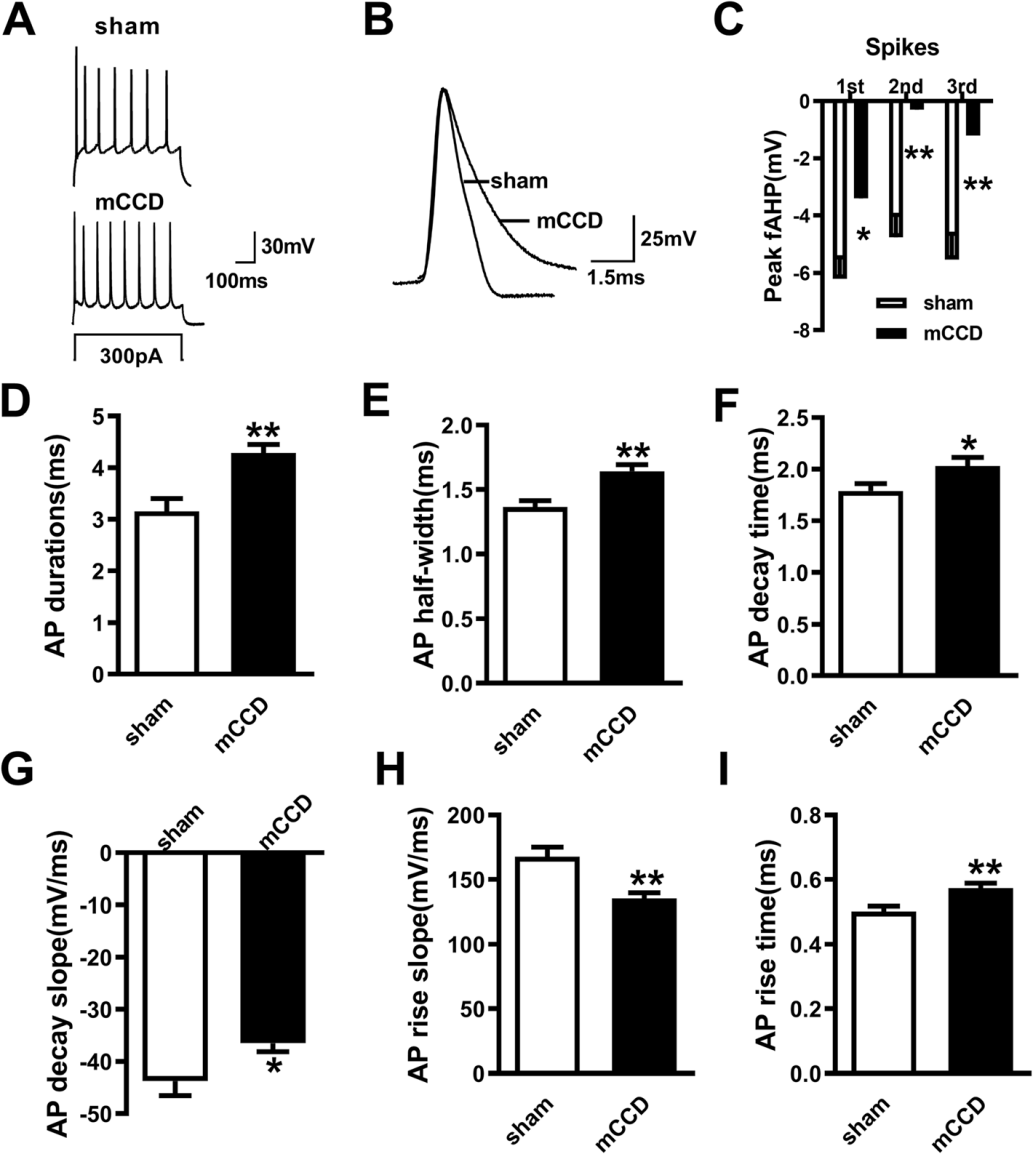
Elevated excitability of pyramidal neurons in the ACC in mCCD rats. **a,** Representative traces showing the firing property in the neurons from ACC of sham and mCCD rats response to a 300 pA depolarizing current injection (500 ms). **b**, Representative recording of the first spike in the neurons from sham and mCCD slices. **c,** The amplitude of fAHP of the first three spikes in a train. **d-i,** The duration (**d**), half width (**e**), decay time (**f**), dacay slope (**g**), rise slope (**h**), rise time (**i**) of the first spike of pyramidal neurons from mCCD and sham rats (mCCD: *n* = 24; sham: n = 35) ^*^p < 0.05, ^**^p < 0.01 compared with that of sham group

To investigate whether the intrinsic properties of ACC pyramidal neurons are altered after mCCD, we studied the firing patterns and the action potential in pyramidal neurons in the layer II or layer III of acutely isolated the ACC slices from mCCD or sham rats. Whole-cell patch clamp recordings were performed on ACC pyramidal neurons on postoperative 7–14 days after mCCD, a time when the maximal behavioral sensitization can be observed. Recorded neurons were identified as pyramidal neurons based on their ability to show spike frequency adaptation in response to prolonged depolarizing-current injection [39]. We compared the passive membrane and the first action potential properties in a train elicited by a 300 pA, 500 ms depolarizing current (Fig. 2a). There were no significant differences in parameters, such as RMP, membrane capacitance (Cm), action potential threshold among ACC neurons between sham and mCCD rats. However, the membrane resistance (Rm), rheobase, action potential amplitude, fAHP peak voltage, duration, half width, decay time, decay slope, rise time and rise slope were significantly changed (Fig. 2 and Table 2). Rm was larger (*p* < 0.05), rheobase was smaller (*p* < 0.05), action potential amplitude was smaller (*p* < 0.05), the fAHP for the first three APs in a train was reduced (Fig. 2c), duration and half-width was significantly increased (Fig. 2d and e), decay time and rise time was longer (Fig. 2f and i), and decay slope and rise slope was slower (Fig. 2g and h) in ACC pyramidal neurons of mCCD rats.

**Table 2:** Intrinsic passive and active properties of ACC pyramidal neurons after mCCD

	sham	mCCD
Resting membrane potential (mV)	−65.32 ± 1.26 (*n* = 38)	−64.79 ± 0.76 (*n* = 49)
R_m_ (mΩ)	75.08 ± 4.01 (*n* = 35)	104.5 ± 10.15 (n = 29)*
C_m_ (pF)	138.4 ± 12.38 (*n* = 35)	168.9 ± 14.21 (*n* = 29)
Rheobase (pA)	129.4 ± 10.66 (*n* = 34)	99.57 ± 5.91 (*n* = 47)*
Action potential threshold (mV)	−31.47 ± 1.29 (n = 34)	−30.02 ± 0.94 (n = 47)
Action potential amplitude (mV)	96.05 ± 2.17 (*n* = 32)	88.82 ± 2.07 (n = 47)*

*p < 0.05, compared with that of sham group

### Effects of NS1619 and paxilline on the excitability of pyramidal neurons in the ACC following mCCD

**Fig. 3:**
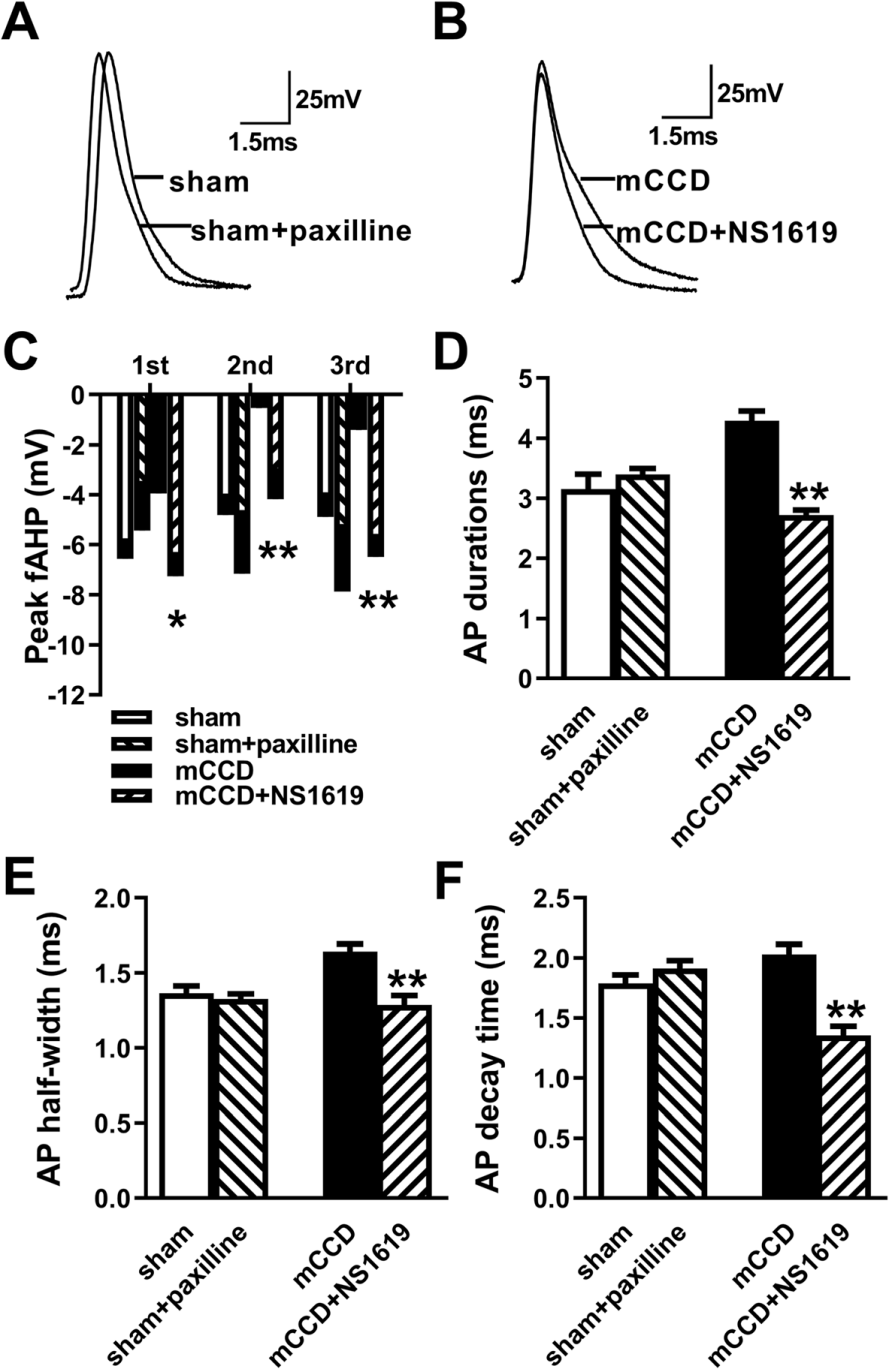
Effects of NS1619 and paxilline on the excitability of pyramidal neurons in the ACC following mCCD. **a**-**b**, Representative recording traces of the first spike in the neurons from sham and mCCD slices in the presence of paxilline or NS1619 respectively. **c**, Histograms show the amplitude of fAHP of the first three spikes from the sham group in the absence and presence of paxilline (10 μM) (sham, *n* = 30, sham+paxilline: *n* = 10), and from the mCCD group in the absence and presence of NS1619 (10 μM) (mCCD: *n* = 48, mCCD+NS1619: *n* = 17).**d-f,** Histograms show AP duration (**d**), AP half-width (**e**) and AP decay time (**f**) from the sham group in the absence and presence of paxilline (sham: n = 30, sham+paxilline: n = 10), and from the mCCD group in the absence and presence of NS1619 (mCCD: n = 48, mCCD+NS1619: n = 17). ^*^p < 0.05, ^**^p < 0.01compared with that of mCCD group

To test whether the role of BK_Ca_ channels in the parameters’ variation of ACC pyramidal neurons in mCCD rats, action potentials were generated in ACC pyramidal neurons in the presence or absence of NS1619 (10 μM). As illustrated in Fig. 3, in mCCD rats, NS1619 significantly increased the peak of fAHP for the first three action potentials in the train elicited by 300 pA, decreased the duration and half-width and increased the dacay time. While paxilline (10 μM), a blocker of BK_Ca_ channel, failed to affect those parameters in the sham rats. The results implicate the functional roles of BK_Ca_ channel in the control of intrinsic excitability in ACC pyramidal neurons after neuropathic pain.

### Reduced BK_Ca_ currents of ACC neurons in mCCD rats

**Fig. 4:**
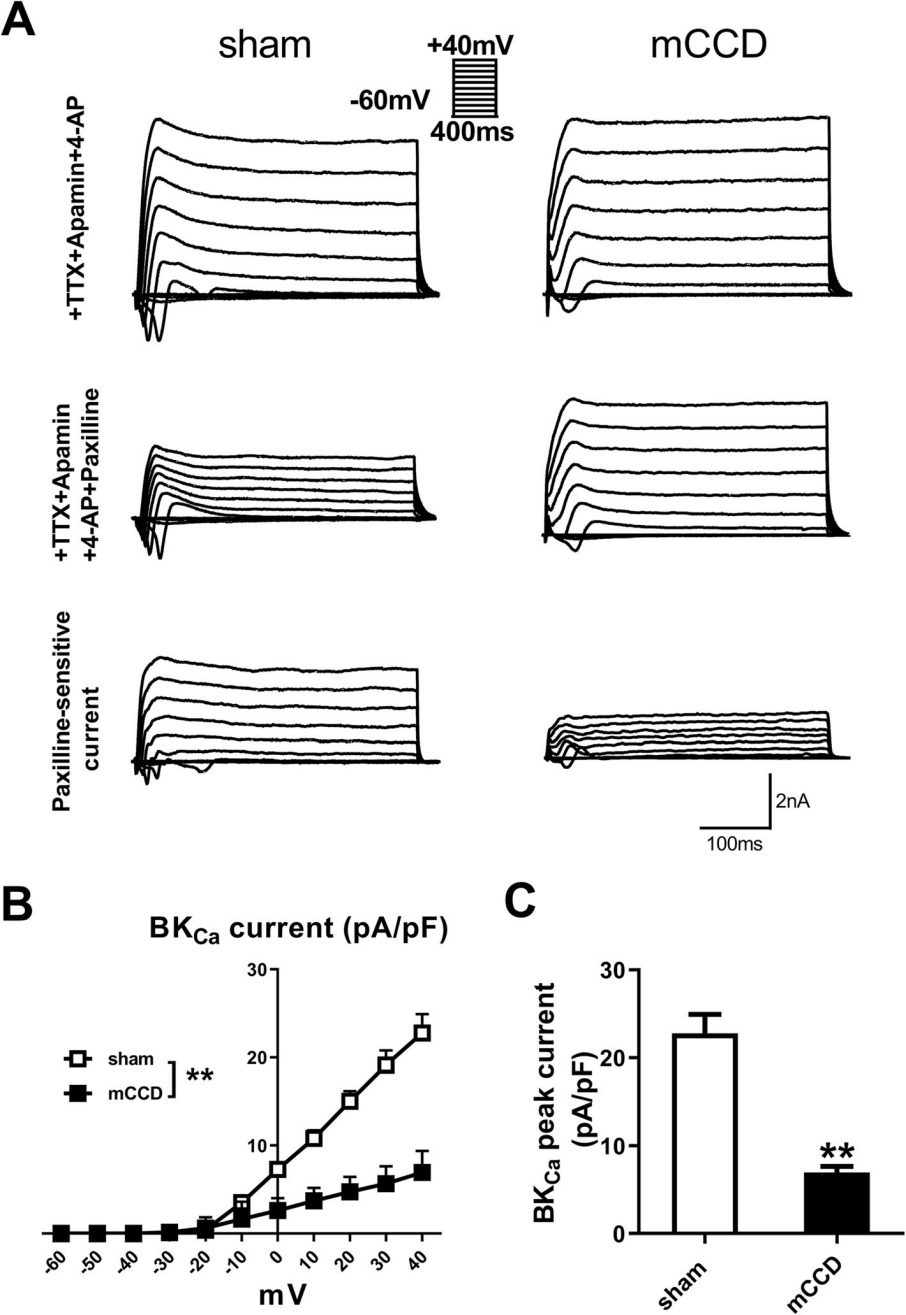
Decreased BK_Ca_ currents of ACC pyramidal neurons from mCCD rats. **a**, Voltage ranges from − 60 to + 40 mV with 10 mV increments, typical recordings of BK_Ca_ currents in pyramidal neurons from sham (left) and mCCD (right) rats, BK_Ca_ currents were isolated with paxilline (10 μM). **b**, The I-V relationship curves showed the differences in the ACC pyramidal neurons from mCCD (*n* = 12) and sham (n = 12) rats. **c**, BK_Ca_ current density at + 40 mV from voltage-clamp recordings of ACC pyramidal neurons from mCCD (n = 12) and sham (n = 12) rats. ^**^p < 0.01 compared with that of sham group

To test BK_Ca_ current of ACC pyramidal neurons in mCCD rats, we performed voltage-clamp recordings. Paxilline (a selective antagonist of BK_Ca_ channel) is often used to confirm the BK current. The membrane potential of recorded neurons was stepped from a holding potential of − 60 mV to + 40 mV in 10 mV increments. We first identified the recorded neurons as pyramidal neurons based on their ability to show spike frequency adaptation in response to 500 ms depolarizing-current injection. The spikes were eliminated by adding the TTX (1 μM), apamin (200 nM) and 4-AP (10 mM) to ACSF to block action potentials, and minimize contamination by small-conductance, calcium-activated K^+^ (SK) and voltage-dependent K^+^ (KV) currents, and then switched to perfusate containing TTX, apamin, 4-AP and paxilline (10 μM) (+TTX + apamin+ 4-AP + Paxilline, wash-in duration: 5 min) to obtain paxilline-sensitive current by subtracting +TTX + apamin+ 4-AP + Paxilline currents from +TTX + apamin+ 4-AP currents. Paxilline-sensitive BK_Ca_ currents were significantly reduced in the mCCD rats compared with those of sham rats (Fig. 4a and b). I-V curve analysis also showed that BK_Ca_ currents were strongly decreased after mCCD (Fig. 4b). The amplitude of currents at the membrane potential of + 40 mV in the mCCD group was significantly reduced compared with that of sham group (Fig. 4c). In page 10, line 36, authors should use same style of °C.

### Up-regulation of BK_Ca_ β4 subunit in the ACC in neuropathic pain

**Fig. 5:**
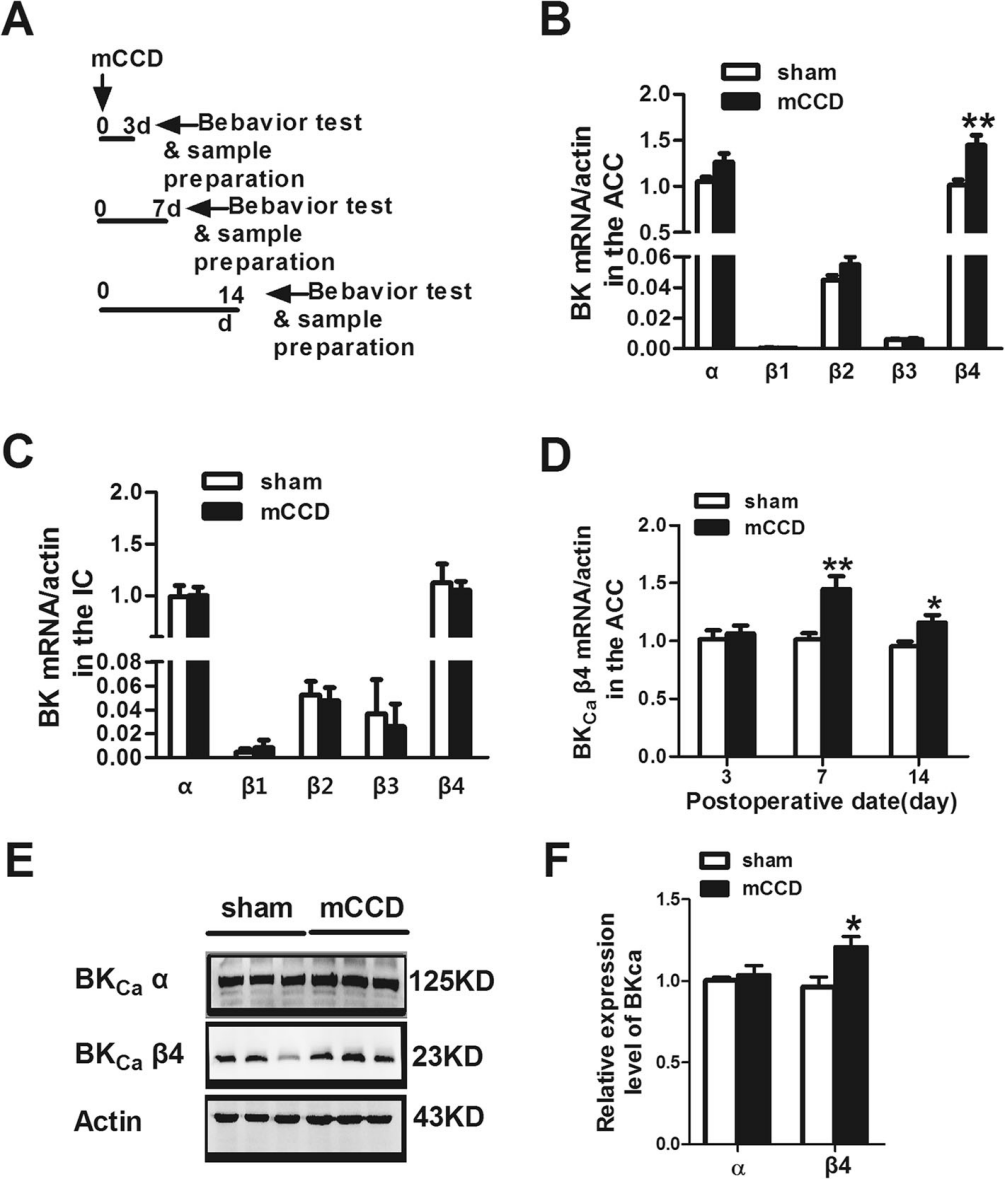
Upregulation of BK_Ca_ β4 subunit protein and mRNA in the ACC during neuropathic pain associated anxiety-like behavior. **a,** The schematic diagram of the behavioral and biochemical experiments. **b,** Quantification of the mRNA levels of BK_Ca_ channel subunits in the ACC between mCCD (n = 7) and sham (*n* = 6) rats on postoperative day 7. **c,** Quantification of the mRNA levels of BK_Ca_ channels in the insular cortex between mCCD (n = 7) and sham (n = 6) rats on postoperative day 7. **d,** Quantification of the mRNA levels of BK_Ca_ β4 subunit in the ACC between mCCD (n = 6) and sham (n = 6) rats on postsurgical day 3, 7, 14. **e,** Representative Western blots for BK_Ca_ α and BK_Ca_ β4 subunit in the ACC obtained on postsurgical day 7. **f,** Quantification of the protein levels of α and β4 subunits in the ACC between mCCD (n = 3) and sham (n = 3) rats on postoperative day 7. ^*^p < 0.05, ^**^p < 0.01 compared with that of sham group

To investigate whether the expression of BK_Ca_ channels is involved in the ACC in neuropathic pain state, we performed RT-PCR and Western blotting to examine the mRNA and protein levels of BK_Ca_ channels (Fig. 5). Our results showed that there were expressed in mRNA level of BK_Ca_ channels in the ACC and the insular cortex on day 7 after the surgery, including α subunit, the dominant subunit of BK_Ca_ channels, and β1–4 subunits (Fig. 5b and c). mRNA expression of BK_Ca_ β4 subunit was remarkably increased in the ACC on postsurgical day 7 (Fig. 5d), while there were no differences in mRNA level of α subunit, the dominant subunit of BK_Ca_ channels, and β1–3 subunits (Fig. 5b) in the ACC on day 7 after surgery. To further test whether the change of BK_Ca_ β4 subunit is region specific for the ACC after neuropathic pain, we also tested the mRNA levels of BK_Ca_ subunits in the insular cortex, another brain region important for pain-related perception. In contrast, no difference of the expression of all subunits of BK_Ca_ channels in the insular cortex was observed on postsurgical days 7 (Fig. 5c).

Moreover, we examined the abundance of BK_Ca_ β4 subunit in the ACC in different times on day 3, 7, 14 after surgery (Fig. 5d). Our results showed that the abundance of BK_Ca_ β4 subunit was significantly increased on postsurgical day 7 and 14, but not on day 3. Similarly, the protein expression of BK_Ca_ β4 subunit in the ACC from mCCD rats was also increased on postsurgical day 7 compared with sham rats (Fig. 5e and f). All these results indicate that the expression of BK_Ca_ β4 subunit is specifically increased in the ACC during neuropathic pain.

**Fig. 6:**
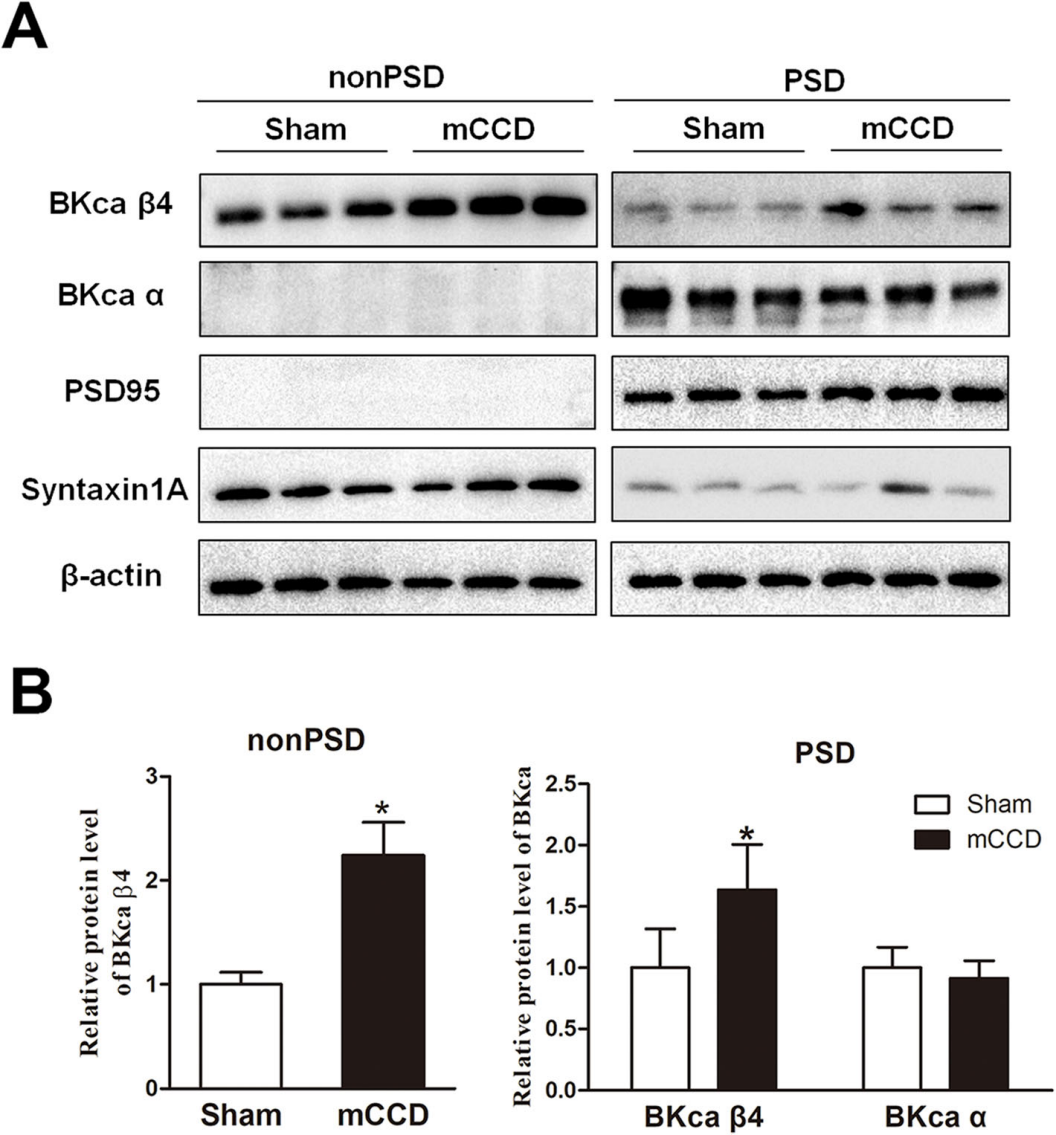
Postsynaptic and presynaptic upregulation of BK_Ca_ β4 subunit protein in the ACC during neuropathic pain associated anxiety-like behavior. **a**, Representative Western blots for PSD 95, syntaxin 1A, BK_Ca_ β4 and BK_Ca_ α subunits in the non-PSD and PSD membrane fractions of the ACC in sham and mCCD rats on postoperative day 14; **b**, BK_Ca_ β4 subunit was significantly enhanced in the PSD and non-PSD fractions of the ACC from mCCD (PSD fraction: 164 ± 37%, n = 6, ^*^p < 0.05; non-PSD fraction: 224 ± 32%, n = 6, ^*^p < 0.05) rats on postoperative day 14 compared with those in sham rats (PSD fraction: 100 ± 32%, n = 6; non-PSD fraction: 100 ± 12%, n = 6). BK_Ca_ α subunit showed no significant change in the PSD fraction between sham (100 ± 17%, n = 6) and mCCD (91.4 ± 14%, n = 6, *p* > 0.05) rats on postoperative day 14

To determine whether postsynaptic or presynaptic BK_Ca_ subunits are involved in neuropathic pain in mCCD rat, we investigated the abundance of BK_Ca_ α and β4 subunits in different subcellular fractions of the ACC on day 14 after surgery (Fig. 6). Our results showed that BK_Ca_ β4 subunit was located at both the extra-synaptic and presynaptic non-PSD, and the synaptic PSD membrane fractions in the ACC, while BK_Ca_ α subunit only predominated at the synaptic PSD membrane fraction in the ACC of sham rats (Fig. 6). We found that the abundance of non-PSD and synaptic PSD BK_Ca_ β4 were significantly increased in the ACC on postsurgical day 14 following mCCD (Fig. 6, non-PSD fraction: sham group, 100 ± 12%, *n* = 6, mCCD group, 224 ± 32%, n = 6, *p < 0.05; PSD fraction: sham group, 100 ± 32%, n = 6, mCCD group, 164 ± 37%, n = 6, *p < 0.05). Moreover, BK_Ca_ α subunit was no significantly changed in the PSD fraction in the ACC of mCCD rats on postoperative day 14 (Fig. 6, sham group, 100 ± 17%, n = 6, mCCD group, 91.4 ± 14%, n = 6, *p* > 0.05). Together, these data suggest that the synaptic and presynaptic BK_Ca_ β4 subunit might be increased in the ACC during neuropathic pain.

### NS1619 reversed the enhanced AMPA receptor-mediated sEPSC frequency of ACC neurons in mCCD rats

**Fig. 7:**
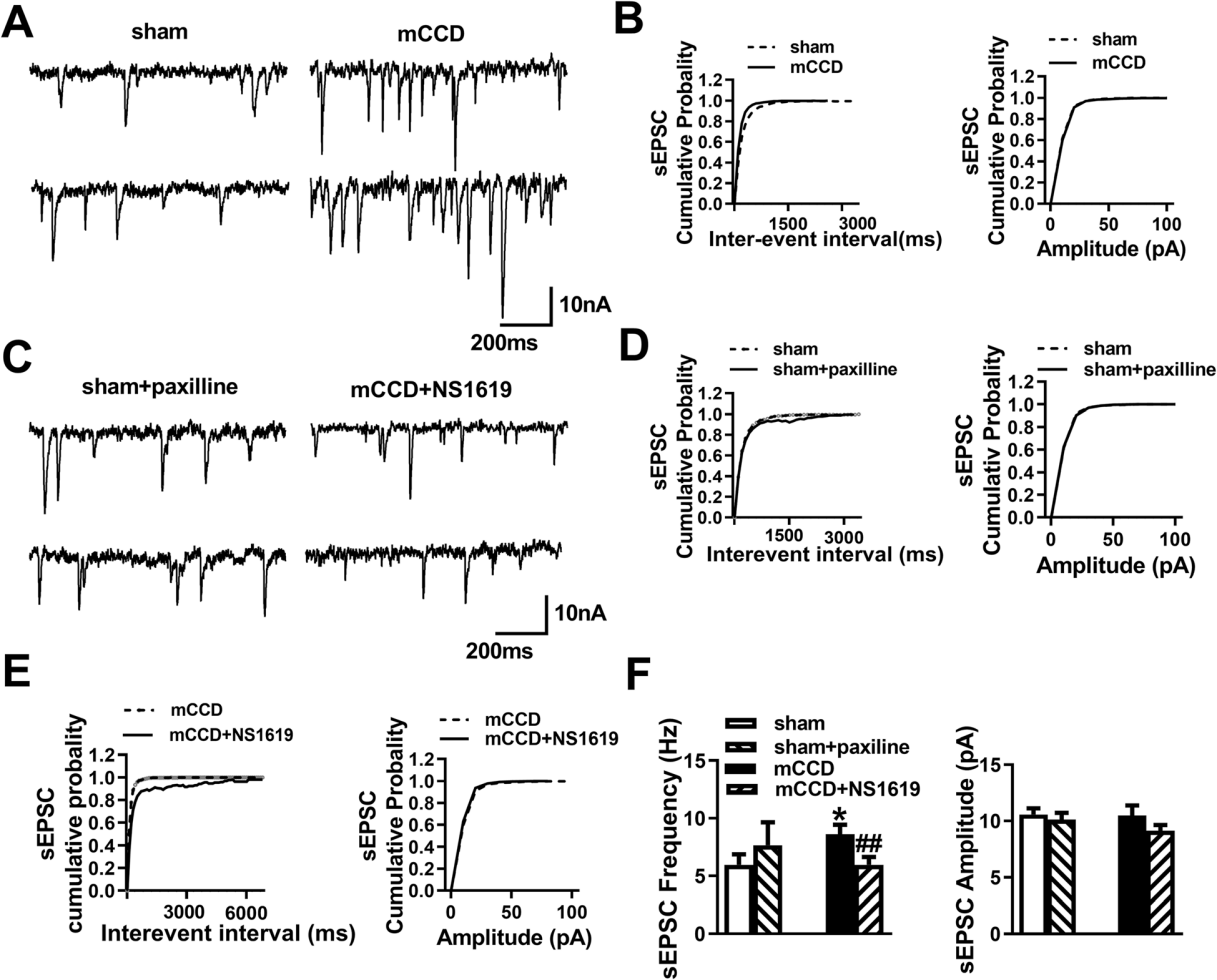
NS1619 decreased the increased AMPA receptor-mediated sEPSC frequency of ACC neurons in mCCD rats. **a,** Representative sEPSCs recorded in ACC pyramidal neuron in slices from sham and mCCD rats at a holding potential of − 60 mV; **b**, Cumulative interevent interval (top) and amplitude (bottom) histograms of sEPSCs; **f**, Summary plots of mean sEPSC peak frequency and amplitude (*n* = 13 neurons for sham and *n* = 16 neurons for mCCD). **c,** Representative traces show AMPA receptor-mediated sEPSCs in the presence of paxilline (10 μM) in sham rats and those in the presence of NS1619 (10 μM) in mCCD rats; **d**, Cumulative interevent interval and amplitude histograms of sEPSCs in the presence of paxilline (10 μM) in sham rats; **e,** Cumulative interevent interval and amplitude histograms of sEPSCs of ACC neurons in the presence of NS1619 in mCCD rats; **f**, Summary plots of sEPSC peak frequency and amplitude in the presence of paxilline (10 μM) in sham rats and those in the presence of NS1619 (10 μM) in mCCD rats(n = 13 neurons for sham and n = 16 neurons for mCCD). ^*^p < 0.05, compared to the value of sham group, ^#^*p* < 0.05, ^##^*p* < 0.01 compared with that of mCCD group

BK_Ca_ channels have been proposed to limit calcium entry and transmitter release by reducing the duration of the presynaptic spike at neurosecretory nerve terminals [47]. It is possible that loss of function of BK_Ca_ channels may lead to the enhancement of basal excitatory synaptic transmission. To explore whether there is any change in the basal excitatory synaptic transmission in the ACC during neuropathic pain, we recorded AMPAR-mediated sEPSCs in pyramidal neurons in the layer II or layer III of acutely isolated ACC slices from mCCD or sham rats on postsurgical day 7 in the presence of AP-5 (50 μM) and picrotoxin (100 μM) (Fig. 7). We found that the sEPSC frequency of ACC neurons was significantly increased in mCCD group than that of sham group, while there is no significant difference in the amplitude of sEPSCs among them (Fig. 7a, b and f). Bath application of NS1619 (10 μM) caused a significant reduction in the sEPSC frequency of ACC neurons in mCCD rats (Fig. 7c, e and f). However, paxilline (10 μM) did not alter either the sEPSC frequency or amplitude of ACC neurons in sham rats (Fig. 7c, d and f). These findings indicate that the excitatory synaptic transmission of the ACC was enhanced and activation of BK_Ca_ channels with NS1619 (10 μM) induced a significant reduction in increased AMPAR-sEPSC frequencies of ACC neurons following mCCD.

### NS1619 increased the paired-pulse ratios (PPR) of ACC neurons in mCCD rats

**Fig. 8:**
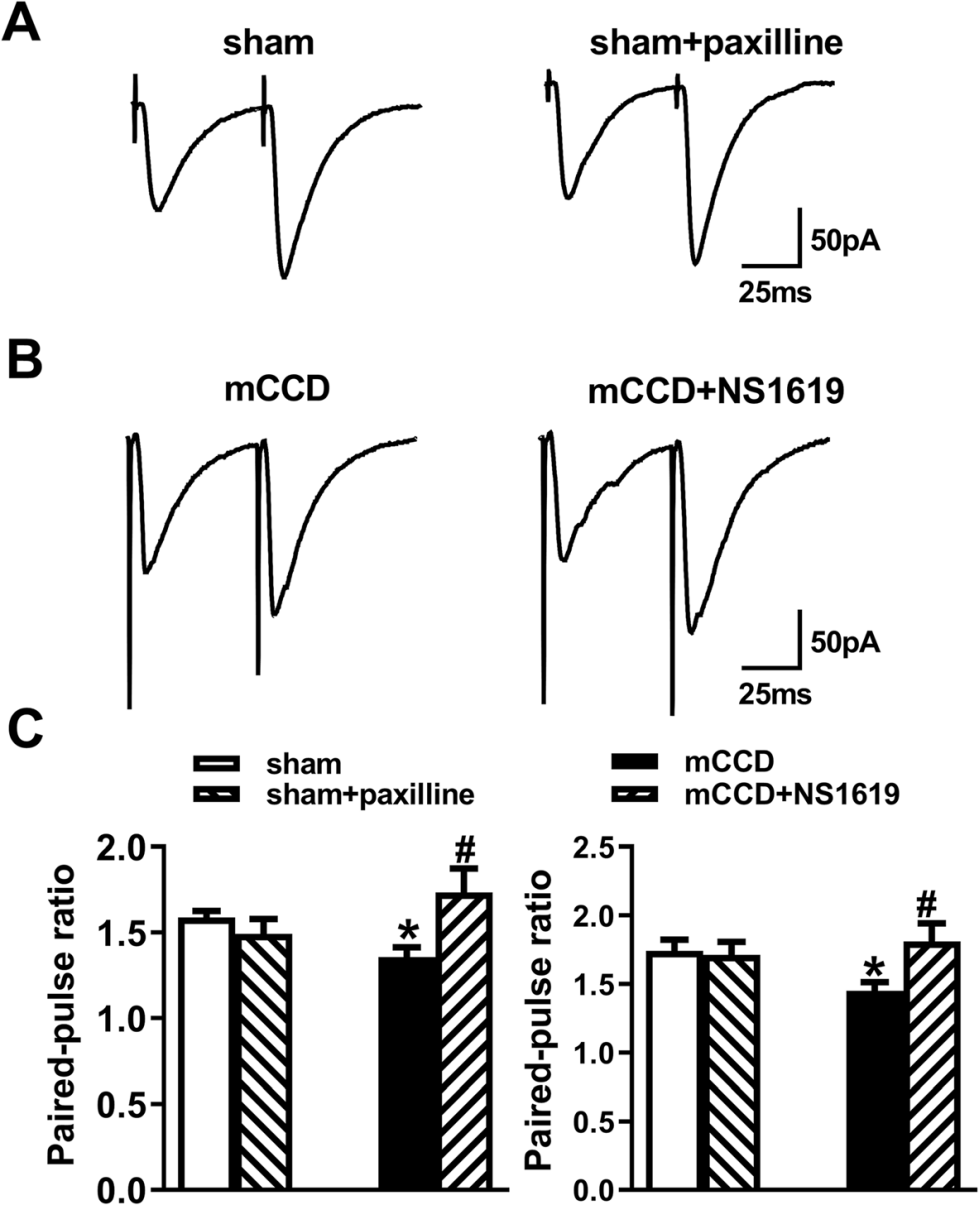
NS1619 enhanced the decreased PPR of ACC neurons in mCCD rats. **a-b,** Representative traces with an interval of 50 ms recorded in layer II/III of the ACC; **a**, PPR (the ratio of EPSC2 / EPSC1) were recorded at intervals of 50 ms from sham rats (n = 6 neurons in the absence or presence of paxilline (10 μM). **b**, PPR were recorded at intervals of 50 ms from mCCD rats (n = 8 neurons in the absence or presence of NS1619 (10 μM). **c**, Summary plots of PPR at intervals of 50 ms from sham and mCCD rats (n = 6 neurons for sham and n = 8 neurons for mCCD), and effects of paxilline and NS1619 on PPR of sham and mCCD rats respectively. ^*^p < 0.05 compared with that of sham group, ^#^*p* < 0.05 compared to that of mCCD group

To validate our hypothesis, we further recorded the PPR of ACC neurons in mCCD rats. PPR is a transient form of plasticity used commonly as a measure of presynaptic function, in which the response to the second stimulus is enhanced as a result of residual calcium in the presynaptic terminal after the first stimulus [48, 49]. Our results showed that PPR at a stimulus interval of 50 ms was significantly reduced in ACC pyramidal neurons from mCCD rats and NS1619 can rescue this reduction (Fig. 8). However, paxilline did not alter the PPR of ACC neurons in sham rats. Together, these results indicate that the enhanced excitatory synaptic transmission is attributable to an increase in the probability of presynaptic neurotransmitter release and BK_Ca_ channels are functionally linked to the synaptic transmission following nerve injury.

### Local activation of BK_Ca_ channels in the ACC reversed neuropathic pain and anxiety-like behaviors

**Fig. 9:**
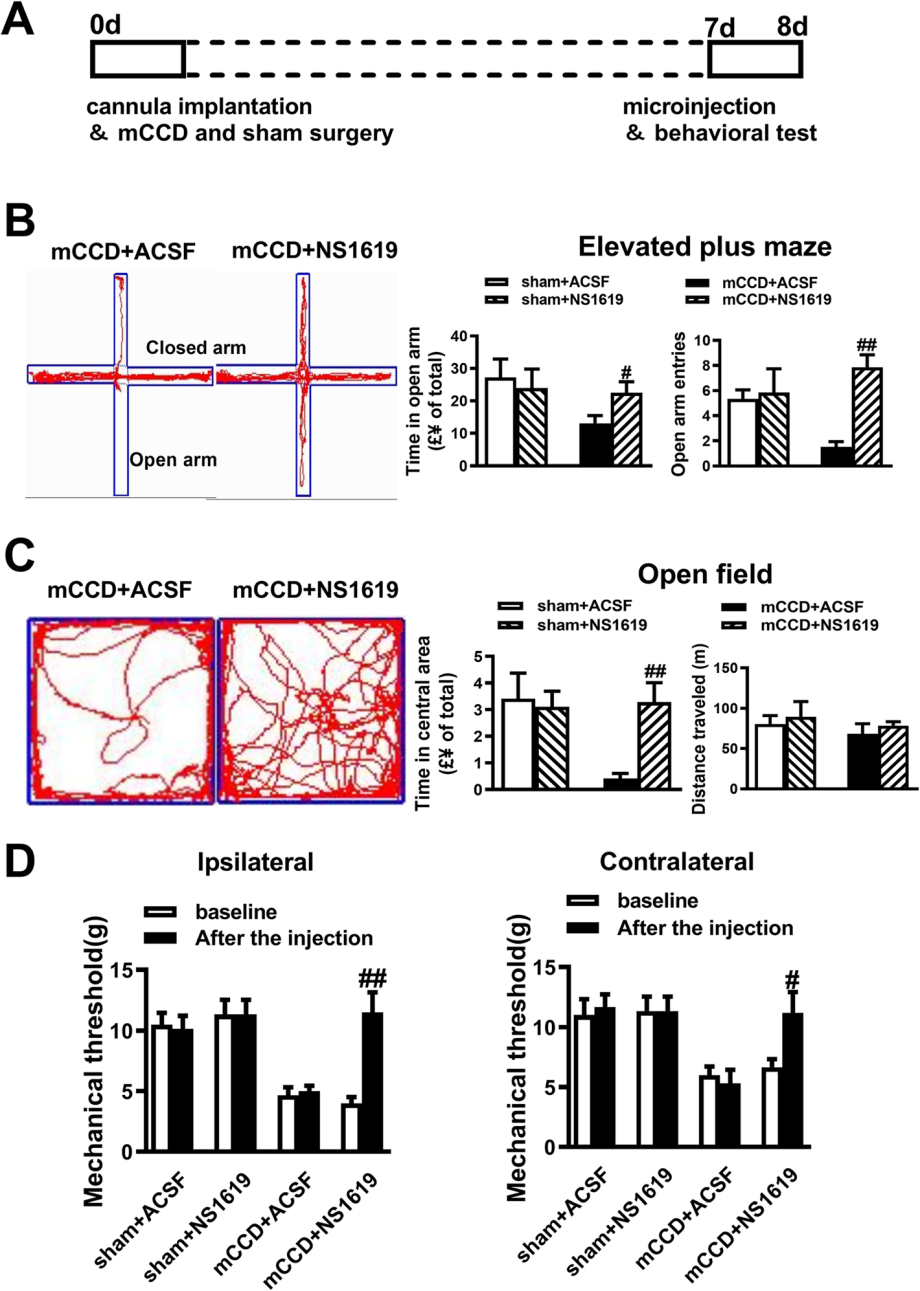
Effects of activating BK_Ca_ channels in the ACC on neuropathic pain and anxiety-like behaviors. **a,** A schematic diagram of microinjection and the behavioral experiment. **b,** Bilateral microinjection of NS1619 (10 μM, 0.5 μL) into the ACC reversed the time in the open arms and the open arm entries in mCCD rats on day 7 after surgery. **c,** Rats infused with NS1619 spent more time in the central areas in open field test in mCCD rats compared with ACSF-treated animals. There was no significant difference in the total distance travelled within the open field for 15 min among mCCD and sham rats (sham + ACSF: n = 6, sham +NS1619: n = 6, mCCD + ACSF: n = 6, mCCD +NS1619: n = 6). **d,** The pain threshold of the injured hind paw (left) and contralateral feet (right), after bilateral microinjection of NS1619 into the ACC on day 7 after surgery. ^#^*p* < 0.05, ^##^*p* < 0.01 compared with that of mCCD with ACSF group

Our results showed that the function of the BK_Ca_ channels were down-regulated in the ACC after neuropathic pain. To test whether ACC BK_Ca_ channels may contribute to behavioral sensitization and anxiety-like behaviors in mCCD rats, we microinjected the BK_Ca_ channels opener NS1619 into the ACC and evaluated the effect of NS1619 on the pain threshold of the injured and contralateral feet and anxiety-like behaviors (Fig. 9a). Bilateral infusion with NS1619 (10 μM, 0.5 μl) reversed neuropathic pain associated anxiety-like behavior in EPM exploration, measured as time in the open arms, between NS1619 and ACSF injected animals (Fig. 9b). Similarly, in the open field test, mCCD rats spent more time in the central areas after treatment with NS1619 compared with those injected with ACSF (Fig. 9c). There was no significant difference in terms of the total distance travelled within the open field for 15mins, indicating that motor coordination and motor function were not changed in all rats examined (Fig. 9c). After bilateral infusion of NS1619 into the ACC on postsurgical day 7, behavioral sensitization of both injured and contralateral hind paws were significantly reversed compared with those of saline microinjection (Fig. 9d). However, NS1619 did not alter the mechanical sensitivity in sham rats compared to a ACSF group (Fig. 9d). This demonstrates that pharmacological activation of BK_Ca_ channels can reverse the effects of neuropathic pain.

## Discussion

The present study provided the strong evidence that mCCD rats exhibited an enhanced anxiety-like behavior, which accompanied bilateral mechanical hypersensitivity. The upregulated expression of the auxiliary BK_Ca_ β4 subunit in the ACC induced the downregulated function of BK_Ca_ channels in the ACC following neuropathic pain. Presynaptic and postsynaptic upregulation of BKβ4 subunit may be involved in increased neuronal excitability and synaptic transmission in the ACC in neuropathic pain state. Local activation of BK_Ca_ channels in the ACC reversed neuropathic pain and anxiety-like behaviors. These results suggest that the upregulated expression of BK_Ca_ β4 subunit in the ACC might contribute to anxiety-like behaviors following neuropathic pain.

### Upregulation of postsynaptic auxiliary BK_Ca_ β4 subunit in the ACC might contribute to anxiety-like behaviors following neuropathic pain

BK_Ca_ channels are known as large single-channel conductance of 100–300 pS among the super family of potassium channels, and dual activation by membrane depolarization and increases in intracellular calcium concentration [50–53]. BK_Ca_ channels play important roles in the regulation of action potentials and excitability in neurons, such as the repolarization of the AP as well as the fAHP [54–56]. Our results showed an enhanced anxiety-like behavior accompanied neuropathic pain in mCCD rats and an increase in the intrinsic excitability of ACC pyramidal neurons of mCCD rats including a decrease of fAHP, rheobase, and action potential amplitude and an increase of Rm, AP duration, half-width and decay time. The increased excitability in ACC neurons of mCCD rats was in line with that of ACC neurons following nerve ligation [40]. The changes of a decrease of fAHP, rheobase, and action potential amplitude and an increase of Rm, AP duration, half-width and decay time strongly indicate a reduction of function and/or expression in BK_Ca_ channels in ACC pyramidal neurons of mCCD rats. In the meantime, NS1619, an agonist of BK_Ca_ channels, significantly rescued the alteration of fAHP and AP duration in ACC pyramidal neurons in the mCCD rat. And paxilline, an inhibitor of BK_Ca_ channels, failed to influence the fAHP peak of ACC pyramidal neurons in the sham rat. All these data confirmed that down-regulated function of BK_Ca_ channels contributed to the alteration of AP repolarization and fAHP in ACC neurons in neuropathic pain state. Consistently, our results further showed that BK_Ca_ current was markedly decreased in ACC neurons of the mCCD rat. These results were also similar to the decrease of BK_Ca_ currents in dorsal root ganglion neurons in neuropathic pain [30, 31, 57], and in CA3 pyramidal neuron of Fmr1 knock-out mice with anxiety-like behavior [58]. In contrast to the down regulation of alpha subunit of BK_Ca_ channels in the lateral amygadala (LA) [32], our molecular biological data showed the up-regulation of mRNA and protein expression of BK_Ca_ β4 subunit were specially increased in the ACC following mCCD. BK_Ca_ channels can be associated with accessory β subunits. Every β subunit has a specific tissue distribution and that they modify channel kinetics as well as their pharmacological properties and the apparent Ca^2+^ sensitivity of the α subunit in different ways [23]. Although previous studies showed that β4 increased BK_Ca_ channel to open [59, 60], β4 subunit could be regarded as an inhibitory neuronal subunit of BK_Ca_ channels [24, 61, 62]. Our results showed that BK currents were decreased in the ACC due to the up-regulation of BK_Ca_ β4 subunit following nerve injury. It is suggested the inhibitory effect of the up-regulation of β4 subunit on the function of BK_Ca_ channels in the ACC in neuropathic pain associated with anxiety-like behavior. In addition, our results further demonstrated that pharmacological enhancement of BK_Ca_ channel function in the ACC reversed neuropathic pain and anxiety-like behaviors. A previous study showed that the activation of BK_Ca_ channels in the LA reversed stress-induced anxiety-like behavior [32]. Recently, it was reported that a selective BK_Ca_ channel opener reversed impaired non-social anxiety-like behavior [63]. Previous studies showed the sensory component of chronic pain remains functionally segregated from its affective and anxiodepressive components at cortical level [1, 64]. However, our data showed the modulation of BK_Ca_ channels might share similar mechanisms in the ACC underlying anxiety-like behaviors accompanied with neuropathic pain.

Membrane-bound calcium-stimulated adenylyl cyclase1 (AC1) is specifically and highly expressed in cortical neurons including the ACC and the insular cortex [49, 65–67]. Recent studies suggest that the surface trafficking of BK_Ca_ channels can be modulated by signaling cascades and assembly with accessory proteins [68], and BK_Ca_ β4 surface trafficking must be post-transcriptionally regulated [69]. Moreover, BK_Ca_ β4 subunit is largely retained in intracellular compartments through an endoplasmic reticulum (ER) retention signal [70, 71]. More recently an imaging research demonstrated that activation of AC reduced BK_Ca_ α surface levels in HEK293 cells with fluorogen activating peptide [68]. These studies indicate that it is very likely that AC1 may modulate the trafficking of BK_Ca_ channels in the ACC in neuropathic pain.

### Upregulation of presynaptic BK_Ca_ β4 subunit in synaptic transmission in the ACC in neuropathic pain state

Modulation of presynaptic AP duration excerts a precise and powerful mechanism to control and regulate neurotransmitter release. AP duration is a critical determinant for transmitter release, controlling the amount of presynaptic calcium influx, which translates electric changes to the release magnitude of neurotransmitters. The AP duration is controlled primarily by the activity of voltage-gated K^+^ channels (VGKCs) [72]. Among VGKCs, BK_Ca_ channels mainly determine AP duration during repetitive activity, owing to their activation being both voltage and calcium regulated in central neurons [55]. For example, BK_Ca_ channels control transmitter release in the synapse [47, 56, 73, 74]. FMRP regulates neurotransmitter release and short-term plasticity through presynaptic BK_Ca_ channels in hippocampal pyramidal neurons [58]. Our data showed that sEPSC frequency was significantly increased in the ACC in mCCD rats. Consistent with our previous results, neuropathic pain causes presynaptic enhancement of the excitatory synaptic transmission in the ACC [49]. And up-regulation of presynaptic BK_Ca_ β4 subunit results in a slower repolarization of action potentials which in turn increase the amount of Ca^2+^ flowing into the cell in the ACC following neuropathic pain. Moreover, an agonist of BK_Ca_ channels decreased the increased AMPA receptor-mediated sEPSC frequency and increased the decreased PPR of ACC neurons of mCCD rats. Activation of BK_Ca_ channel function in the ACC reversed neuropathic pain and anxiety-like behaviors.

Taken together, these results indicate that the upregulation of presynaptic BK_Ca_ β4 subunit might also increase basic synaptic transmission in a presynaptic modulation. BK_Ca_ channels mediated neuropathic pain induced anxiety-like behaviors via the increase of presynaptic neurotransmitter release.

## Conclusions

In summary, our study indicated presynaptic and postsynaptic up-regulation of BK_Ca_ β4 subunit in neuronal excitability and synaptic transmission in the ACC in neuropathic pain state. In physiological states, BK_Ca_ β4 subunit is expressed in both presynaptic and postsynaptic neurons, the opening of BK_Ca_ channels allows rapid efflux of potassium ions, which effectively hyperpolarizes membrane potential. In neuropathic pain states, the upregulation of postsynaptic BK_Ca_ β4 subunit induces neuronal activation easily due to the loss function of BK_Ca_ channels. In the meantime, the upregulation of presynaptic BK_Ca_ β4 subunit results in a more slow repolarization of action potentials which in turn increases the amount of Ca^2+^ flowing into the cell. Thus BK_Ca_ channels enhance the transmitter release in the synapse in the ACC in neuropathic pain state. All these data provided evidence that BK_Ca_ pathway has to be explored as a new interesting therapeutic target for neuropathic pain-associated anxiety. Therefore, our results suggested that BK_Ca_ channel agonist might be a potential candidate for pain-related anxiety treatment because it suppresses neuronal hyperexcitability and decreases neurotransmitter release at the synapse in the ACC following neuropathic pain.

## Data Availability

On behalf of all authors, we declare that the data and material in the manuscript are original and the manuscript is not under consideration for publication elsewhere. In addition, none of the manuscript contents has been previously published.
